# USING THE MOCA-SA WITH SPANISH-SPEAKING OLDER ADULTS: STRENGTHS, LIMITATIONS, AND CORRECTIONS

**DOI:** 10.1093/geroni/igad104.0233

**Published:** 2023-12-21

**Authors:** Lissette Piedra, James Iveniuk, Kelly Pudelek, Melissa Howe, David Marquez

**Affiliations:** University of Illinois at Urbana-Champaign, Urbana, Illinois, United States; NORC at the University of Chicago, Chicago, Illinois, United States; NORC at the University of Chicago, Chicago, Illinois, United States; NORC at the University of Chicago, Chicago, Illinois, United States; University of Illinois Chicago, Chicago, Illinois, United States

## Abstract

Exploratory factor analyses using Round 2 and 3 data reveal discrepancies in the MoCA scores of English and Spanish speakers. In this paper, we describe the strengths and limitations of using NSHAP’s state-of-art MoCA measure with Spanish speakers and propose a solution to the discrepancies observed between the two linguistic groups. To arrive at our remedy, we calculated modification indexes of Spanish language MoCA items to identify items that remained consistent across ethnoracial language groups. From these items, we constructed an abbreviated MoCA-SAA (Spanish version) that compared favorably with the long form MoCA-SA across the different grouping and showed predicative validity with consequential outcomes associated with cognitive decline.

